# Important role of the government in reducing pesticide use and risk sustainably in Thailand: Current situation and recommendations

**DOI:** 10.3389/fpubh.2023.1141142

**Published:** 2023-03-03

**Authors:** Ratana Sapbamrer, Amornphat Kitro, Jinjuta Panumasvivat, Pheerasak Assavanopakun

**Affiliations:** Department of Community Medicine, Faculty of Medicine, Chiang Mai University, Chiang Mai, Thailand

**Keywords:** pesticide, Thailand, policy, legislation, strategy, government

## Abstract

Agriculture is an important aspect of Thailand's GDP and development. It ranks as the 8th largest exporter in the world, however, pesticide use associated with the agriculture is ranked 18th in the world and 5th in Asia. Previous studies have clearly stated that pesticides are a threat to human health and the environment. The government is now making efforts to address pesticide use and the health impacts of pesticide use, however, these efforts are still in need of completion. This paper aims to summarize: (1) the current situation with regard to pesticide use in Thailand; (2) the current situation with regard to pesticide poisoning in Thailand; and (3) important role of government in reducing pesticide use and risk sustainably in Thailand: current situation and recommendations. This article suggests that government is a significant driver for reducing pesticide use and risk sustainably and the establishment of push and pull policies, legislation, and strategies. The government needs to strictly adhere to international conventions. Introducing a pesticide tax is essential to reduce redundant pesticide use. Updating of the current act, specific regulations with regard to pesticides and strict enforcement are urgently required. Licensing pesticide applicators should be implemented continuously. Promotion of alternative pest management is needed by supportive production inputs, technology, and markets as well as the development of a monitoring and certification system. Educating consumers on how to choose safe agricultural products and reduce risk from pesticide residues in the products is also necessary. All approaches should be implemented simultaneously and instantly. Importantly, the government needs to cooperate with agricultural sectors, health and environmental sectors, private sectors, as well as food industries to tackle complicate issues in a sustainable manner and lower pesticide use and risk sustainably in Thailand.

## 1. Introduction

**Figure 1:**
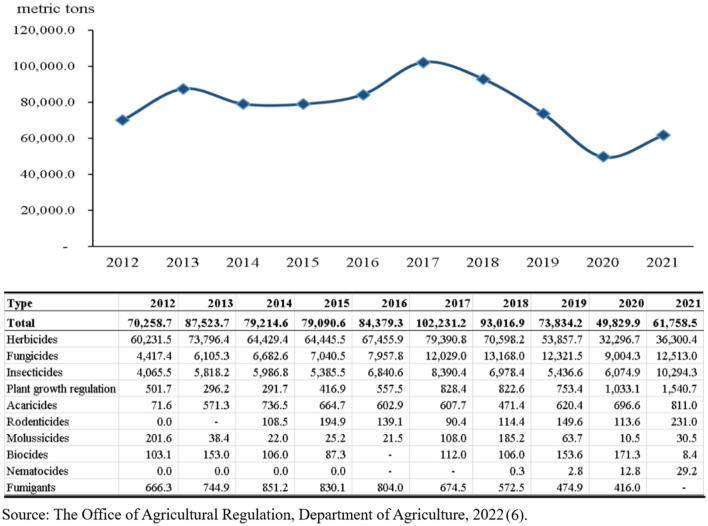
The amounts of pesticides imported into Thailand from 2012 to 2021.

Agriculture is a major part of Thailand's development. The Gross Domestic Product (GDP) contribution from agricultural sector is ~40,398 million US Dollars and the forecasted GDP growth from this sector is about 2.2% (1). Thailand is the 8th largest exporter in the world, with rice, rubber, and cassava being the biggest contributors (2). Pesticide use for agriculture in Thailand is the 18th in the world, and the 5th in Asia, pesticide use being about 1.66 kilograms per hectare of cropland (3–5). The trend of pesticide imports imported into Thailand from 2012 to 2021 tends to fluctuate, with the lowest amounts imported in 2020, and a gradual increase in 2021 (Figure 1) (6). It's possible that the COVID-19 pandemic had an impact on the daily and work lives of farmers. Lockdown and other measures during the COVID-19 pandemic have potentially threatened agricultural production systems, crop yields, and agrochemical logistical problems. Closure of restaurants, food shops, and hotels also influence crop demand, resulting in the reduction of crop yields and pesticide use (7).

**Figure 2:**
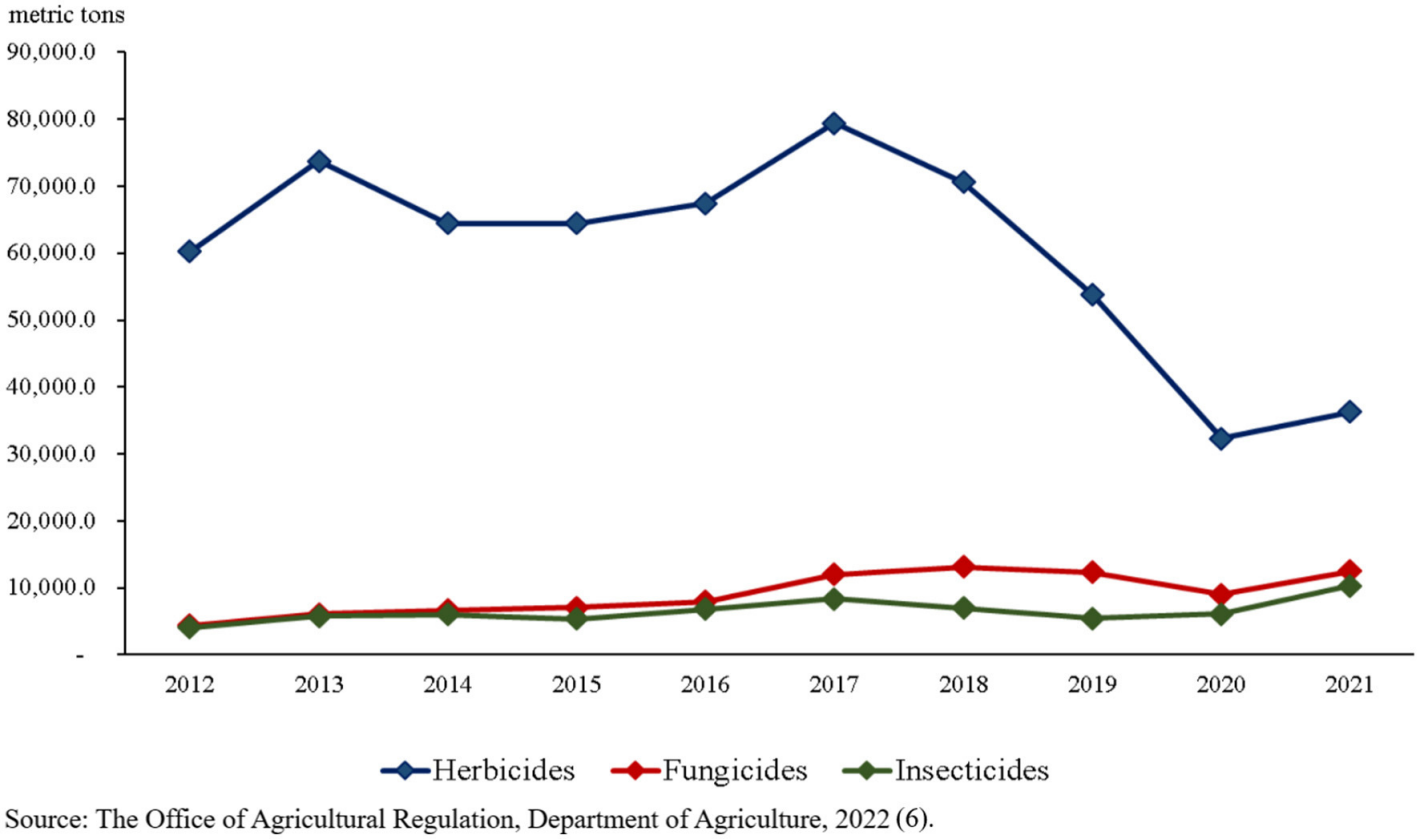
Top three pesticides imported into Thailand from 2012 to 2021.

Regarding type of pesticide use in Thailand, herbicide, fungicides, and insecticides are the top three pesticides used for agriculture purposes (Figure 2). In 2021, herbicides were the most significant import into Thailand (36,300.4 metric tons), followed by fungicides (12,513 tons) and insecticides (10,294.3 metric tons), respectively. Herbicide import trends are declining, whereas fungicide and insecticide import trends are increasing (6). The declining trend for herbicides might be due to the announcement made by the Ministry of Agriculture in 2019 to limit the use of paraquat, and glyphosate. These two herbicides are the major herbicides used for controlling weeds. However, the amounts of imported herbicides are still the largest when compared to fungicides and insecticides (8).

**Figure 3:**
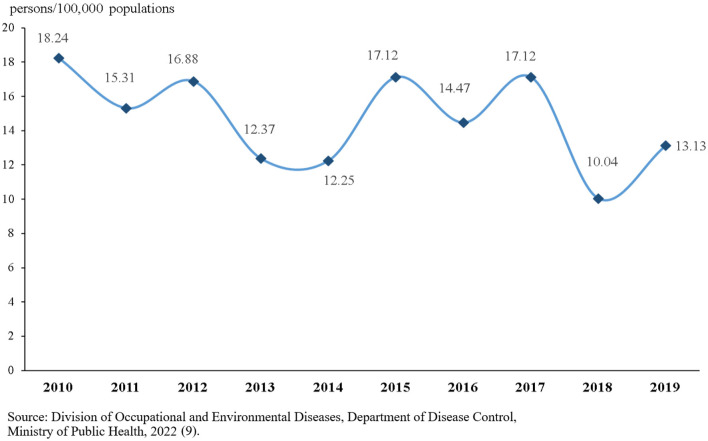
Morbidity rate (person/100,000 populations) of reported pesticide poisoning cases in Thailand from 2012 to 2019.

The Division of Occupational and Environmental Diseases, Department of Disease Control, Ministry of Public Health is responsible for reporting the number of cases of pesticide poisoning annually in Thailand. The highest numbers of cases reported are those resulting from insecticide poisoning. The highest number of reported cases was in 2010, whereas the lowest number was reported in 2018. The reported case trends fluctuate, but they tend to decline (Figure 3) (9).

In reality, however, the reported cases were lower than the actual cases of pesticide poisoning. It is possible that health care services did not enter the code for pesticide poisoning in the International Classification of Diseases and Related Health Problems 10th Revision (ICD-10). Health care services have a duty to enter codes of disease data classified by the ICD-10. The codes for pesticide poisoning are T60.0 (organophosphates and carbamate insecticides), T60.1 (halogenated insecticides), T60.2 (other insecticides), T60.3 (herbicides and fungicides), T60.4 (rodenticides), and T60.8 (other pesticides). However, when it comes to the actual practicality of reporting, farmers and farmworkers usually reside in rural areas and use health services from primary healthcare services in their village (so called “Subdistrict Health Promotion hospitals”) when they become ill. Health care staff at the primary healthcare service may be unable to diagnose pesticide poisoning because the symptoms of pesticide poisoning are non-specific. The common symptoms of pesticide poisoning being headache, dizziness, vomiting, cramp, nausea, wheeze, fatigue, breathlessness, rash, muscle problem, etc. Therefore, the staff potentially did not enter the code of pesticide poisoning in the ICD-10. Educating and training for primary health care staff about diagnosis and treatment of pesticide poisoning is necessary (10). Another possibility is that people who had mild symptoms of pesticide poisoning did not seek treatment at hospitals, resulting in some cases missing from the ICD-10 (11, 12). Furthermore, most farmers and migrant domestic workers were classified into informal sectors; therefore, they are not protected job security, wages, and occupational safety and health under labor and occupational safety and health regulations (12). As a result, accessibility of farmers and migrant domestic workers to health care services may be limited even if they become ill from health-related pesticide poisoning. Although Control of Occupational Diseases and Environmental Diseases Act, B.E. 2562 (2019) has been issued and covers the informal sectors, this Act doesn't yet define criteria and methods of health surveillance system for diseases related pesticide exposure (13). Underreported pesticide poisoning cases are also influenced by the health surveillance system. The information in health surveillance system can only provide the number and distribution of pesticide poisoning cases, but not the risks of pesticide exposure and causes of pesticide poisoning (14). The government is now making efforts to address pesticide use and the health impacts of pesticide use, however, these efforts are still in need of completion. Therefore, this article provides a current role and situation of Thailand's government in reducing pesticide use and risk, and propose recommendations in order to promote sustainable development in Thailand.

## 2. Important role of government in sustainably reducing pesticide use and risk in Thailand

**Figure 4:**
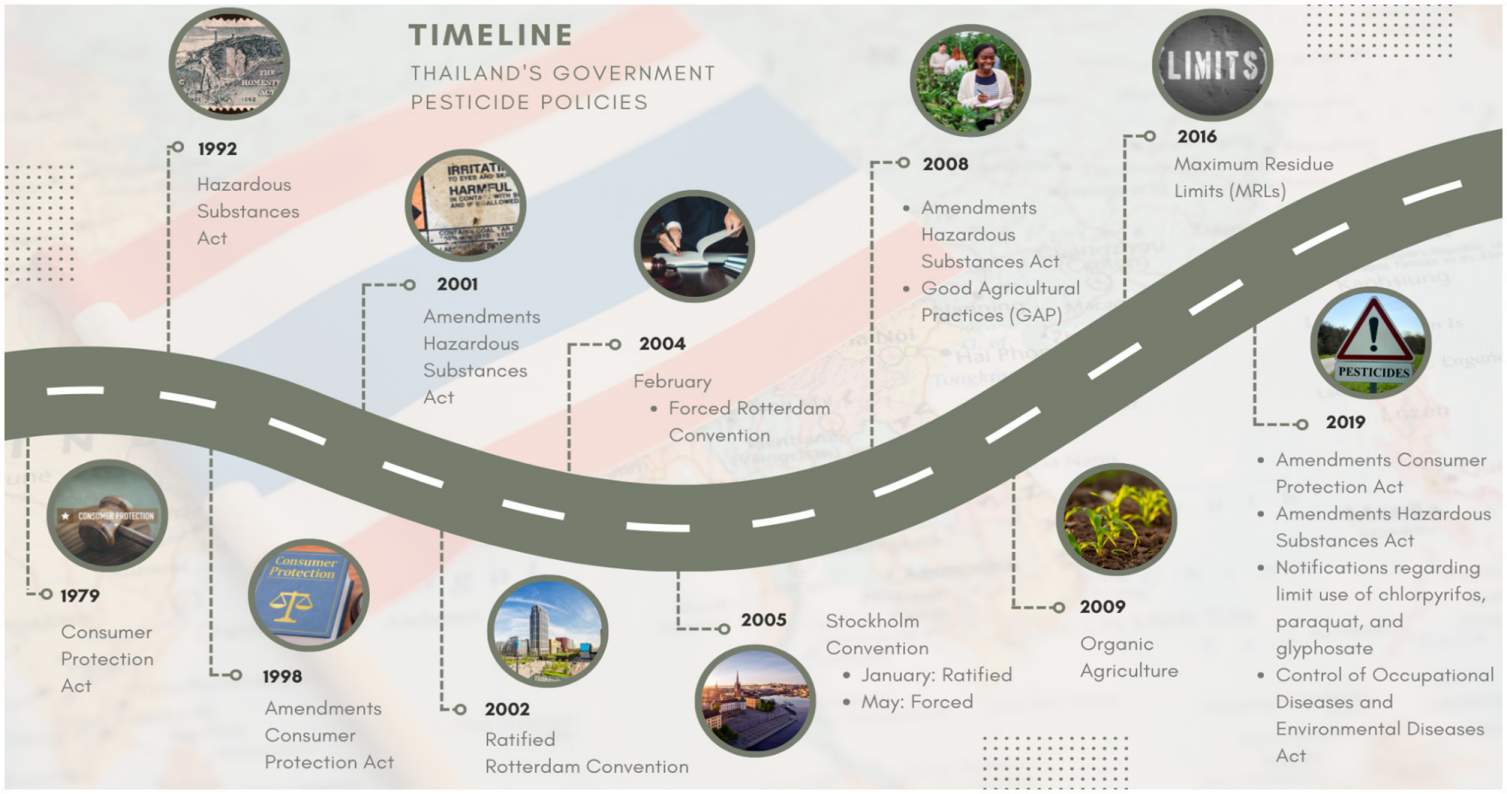
Pesticide policies of Thailand's government.

To ensure reduction of pesticide use and risk sustainably in Thailand, the government plays a vital role in achieving that goal. Thailand's government has several policies in reducing pesticide use and risk (Figure 4). However, the implementation of the government nowadays is still incomplete. Therefore, the government should establish push and pull policies, legislation, and strategies, all approaches needing to be implemented simultaneously. Important approaches needed include a common approach by The Parties of International Conventions, the introduction of incentive and pesticide tax policies, strict enforcement of legislative measures, measures to regulate pesticide residues in agricultural products, licensing of pesticide applicators, promotion of alternative pest management, promotion of PPE use during pesticide application, strengthening knowledge and raising awareness of pesticide issues (Table 1).

**Table 1:** Important role of Thailand's government: Current situation and recommendations.

**Important role of the government**	**Current situation in Thailand**	**Recommendations**
The parties of international conventions	Unable to access the information of non-members of industrial associations	Strengthen law enforcement through the Hazardous Substances Act, B.E.2535 (1992)
Introducing incentive and pesticide tax policies	No existence of pesticide taxes and incentive policies	Revise the pesticide tax system and develop incentive policies
Strict enforcement of legislative measures	The current Act does not regulate specific pesticides in agriculture	Update specific regulations for pesticides in agriculture
	Illegal use of pesticides in agriculture	Enforce strictly the regulations
Measures to regulate pesticide residues in agricultural products	No main agency for monitoring pesticide residues in agricultural products	Establish an overarching organization for monitoring pesticide residues in agricultural products
Licensing of pesticide applicators	Licensing of pesticide applicators for limit use of chlorpyrifos, paraquat, and glyphosate	Licensing of pesticide applicators for all pesticide application
Promotion of alternative pest management	Lack of knowledge in alternative pest management	Continual training in alternative pest management for agricultural officers and farmers
	Lack of production inputs and a scarcity of markets	Support production inputs, technology, and markets
	Complicated and time-consuming monitoring and certification systems	Develop a monitoring and certification system
	Several labels of certificates attached to the agricultural products	Educate consumers about various agricultural product labels through the media, schools, and communities
Promotion of PPE use during pesticide application	Rarely use of advanced PPE among farmers due to tropical climatic conditions, uncomfortable when working, poverty, unavailability of PPE, and high cost of PPE	Educate farmers about pesticide safety practices through a life-long training program
		Develop incentive policies to make PPE more available to farmers
Strengthening knowledge and raising awareness of pesticide issues	Lack of knowledge and awareness of pesticide issues among agricultural extension staffs and farmers	Continual training in agricultural extension staffs and farmers to keep up with the changing situation
	Existing agricultural extension operation is rather complicated.	Reinforce the function of agricultural extensions by decentralizing to local governments
	Lack of knowledge and awareness of safe food among consumers	Provide information regarding alternatives for cleaning vegetables and fruits in during food preparation
		Encourage consumers to choose and pay for safe food

### 2.1. The parties of international conventions

The Rotterdam Convention and the Stockholm Convention are multilateral treaties related to the ban and restriction of pesticide use. The Rotterdam Convention is a treaty to promote shared responsibility and efforts among all parties in the international trade of certain hazardous chemicals. The Convention also facilitates information exchange regarding the characteristics of hazardous chemicals, providing national decision making on their import and export, and disseminating their decisions. The ultimate goal of the Convention is to protect both the environment and human health from potentially hazardous chemicals. The Convention came into force on February 24, 2004. The Convention covers 52 chemicals (including 35 pesticides, 16 industrial chemicals, and 1 in both pesticide and industrial chemical categories) that have been banned and severely restricted by two or more Parties and which have been notified by Parties for inclusion in the Prior Informed Consent procedure. There are 165 Parties in this Convention (15).

The Stockholm Convention focusing on persistent organic pollutants (POPs) is an international environmental treaty to eliminate or restrict the production and use of POPs. The goal of this Convention is to protect the environment and human health from POPs. The Convention came into force on 17 May 2004. It covers 35 POPS which can be categorized into three groups, specifically pesticide POPs, industrial POPs, and unintentional production of POPs (16, 17).

Thailand ratified the Rotterdam Convention on 19 February 2002 and it came into force on 24 February 2004. The Stockholm Convention was ratified on 31 January 2005 and came into force on 1 May 2005. There are several benefits to Parties of the Conventions. The Conventions make informed decisions and raise awareness of national bans and severe restrictions on hazardous chemicals with other Parties. They also share responsibility among the Parties to avoid import and export of certain hazardous chemicals. In the case of risk management, exporting Parties provide export notification, labels, and safety information data sheets to confirm dangers of the chemicals to importing Parties. In addition, the Conventions manage networks among designated national authorities to exchange knowledge and experience in the implementation of the Convention. They also provide assistance in technical and economic matters to the Parties in transition. In summary, the Conventions help the Parties to protect the environment and human health from hazardous chemicals.

All chemicals listed in the Rotterdam Convention and the Stockholm Convention come under The Hazardous Substances Act, B.E.2535 (1992). This Act regulates hazardous chemicals across all activities including registration, licensing, and monitoring. However, non-members of industrial associations are unable to access the information. As a result, the strengthening of law enforcement and raising awareness are urgently needed (18, 19).

### 2.2. Introducing incentive and pesticide tax policies

There are several incentive policies in place for reducing pesticide use in some countries. For example, funding and other types of support are available for farmers who control pests by using organic agriculture, integrated pest management, bio-control, natural pesticides, or other non-pesticide methods to change cultivation practices. Policies to encourage the use of pesticides which are manufactured domestically and are less hazardous should be applied through a pesticide tax. Pesticide tax should be implemented on dealers, traders, and retailers who sell pesticides that are imported and have high toxicity level. Whereas, the pesticide tax should be decreased for pesticides that are manufactured domestically and have a low toxicity level. Furthermore, the income from the tax should be used for the support of farmers who use alternative non-pesticide methods, and also to enhance relevant knowledge through research. The scope of research needed includes long-term effects of pesticide exposure, external costs of pesticide use, design of farming systems based on agro-ecology, and alternative crop protection methods (20). The goal of pesticide tax policy is to reduce redundant pesticide use in agriculture while also increasing awareness and social responsibility in the agricultural sector (21).

In Thailand, pesticide tax and incentive policies do not exist. In contrast, the Revenue Department exempts pesticide traders and dealers from paying value added tax (VAT) and in addition, the Customs Department reduces the tax rate of insecticides from 30 to 10% (22). With regard to other related equipment, farmers have to pay 7% VAT for buying mowing machines, tractors, or machines for controlling pests and weeds (23). Lianjamroon (23) also suggested that Thailand pesticide tax charge should be ~12.5–50% of pesticide price depending on the levels of toxicity. The government should utilize the tax revenue to fund the programme aimed at lowering pesticide use. Additionally, a portion of the tax revenue should be used to support incentive policies, like those that promote alternative pest control and food safety research. Therefore, we propose that the Thai government should revise the pesticide tax system and develop incentive policies in order to reduce unnecessary pesticide use in agriculture and promote alternative environmentally friendly and health respecting pest control methods.

### 2.3. Strict enforcement of legislative measures

The primary issues for pesticide management in Thailand are a lack of severe enforcement of the legislation, a lack of accountable agencies, a lack of measures to restrict pesticide advertisement, as well as irresponsible pesticide retailers and dealers (23). Hazardous chemicals which include pesticides are regulated by the Hazardous Substances Act, B.E.2535 (1992), amendments B.E.2544 (2001), B.E.2551 (2008), and B.E.2562 (2019). This Act is enacted for the control of hazardous chemicals across all activities, including registration, licensing, and monitoring. The main tasks are to select safe chemical use in Thailand, have permits for chemicals for import, export and manufacturing, and control product quality in the market after registration (19). The Department of Agriculture, Ministry of Agriculture and Cooperatives is responsible for controlling law enforcement regarding pesticides use in agriculture. However, the existing Act covers all hazardous chemicals, resulting in broad procedures for the control of hazardous chemicals rather than only pesticides. The Act does not regulate specific pesticides in agriculture. In addition, the Act does not cover unintentional consideration of end-use, and handling of pesticides after point of sale (24). Therefore, specific regulations for pesticides in agriculture are required to facilitate effective law enforcement (25).

Importantly, different authorities are responsible for registration and banning of pesticides in Thailand. Department of Agriculture is responsible for the registration, whereas Hazardous Substance Committee chaired by Permanent Secretary of Ministry of Industry is responsible for banning pesticides. Additionally, banning process is extremely time-consuming, and based on available information from International Agency for Research Cancer (IARC), International Programme on Chemical Safety (IPCS), and decision guidance documents (DGDs). However, the majority of pesticide banning did not take into account the current situation regarding the risks and uses of pesticides in Thailand. Therefore, the registration and banning of pesticide processes need to be improved (26).

Illegal use of pesticides in agriculture is also a major problem in Thailand. At present, Thailand prohibits the use of 111 pesticides; however, the banned pesticides are still frequently detected in agricultural products. A study by Sapbamrer and Hongsibsong (27) found monocrotophos, dicrotophos, and parathion-methyl in vegetable samples despite these pesticides being banned. The farmers may be seduced into using these due to their effectiveness in pest management, high demand from users, illegal stock held by traders, and smuggling across the border of Thailand (28). Therefore, strict enforcement of the regulations is urgent in the drive to address the problem of illegal use of pesticides.

### 2.4. Measures to regulate pesticide residues in agricultural products

The National Bureau of Agricultural Commodity and Food Standards, Ministry of Agriculture and Cooperatives issued a revised announcement entitled: “Thai agricultural standards, pesticide residues: Maximum Residue Limits (MRLs) (TAS 9002-2016)” in 2016. There are 56 pesticides which are listed in the MRLs in agricultural products. This notification is a tool for ensuring that agricultural products meet quality standards, and that the health of consumers is protected (24, 29). Consumer Protection Act, B.E. 2522 (1979), amendments B.E.2541 (1998), and B.E.2551 (2562), is also enacted for protecting the consumers by regulating the safety of goods and services (30).

Several organizations offer a service to analyze pesticide residues in food, including The Department of Medical Sciences in Ministry of Public Health, The Department of Research and Development in Agricultural Inputs in Ministry of Agriculture and Cooperatives, and academic institutions. However, no main agency is responsible for overseeing the monitoring of pesticide residues in agricultural products in markets and supermarkets (25). In addition, these notifications are revised every 5–10 years, resulting in it frequently being out to date when it comes to protecting the health of consumers (24). Therefore, the government needs to establish an overarching organization for monitoring pesticide residues in agricultural products continuously, solely in markets and supermarkets. Importantly, pesticide residues above MRLs cause a potential acute health effects, whereas the residues below MRLs may cause chronic health effects. Additionally, the assessment for non-cancer health risk, according to estimated daily intake (EDI) and hazard quotient (HQ) indicates potential threat to children' heath and alarming for adults (31, 32). As a result, notifications for setting new MRLs recommendation should be updated frequently.

### 2.5. Licensing of pesticide applicators

Over the last decade, the Thai public has become increasingly concerned about hazardous pesticides and health and the environment, in particular those associated with chlorpyrifos, paraquat, and glyphosate. In 2019, the Ministry of Agriculture and Cooperatives announced five notifications regarding limitation of use of chlorpyrifos, paraquat, and glyphosate. Measures to limit and ensure safe use of these three pesticides for farmers and pesticide applicators are as follows: (1) register licensed farmers with the Department of Agricultural Extension; (2) pass the training provided by the Department of Agriculture and carry out re-training every 3 years; (3) show an identification card before purchasing these pesticides; and record the quantity of pesticides purchased according to types of crop and crop area; (4) wear PPE during mixing and spraying pesticides; (5) use appropriate spraying equipment; (6) prevent the use of these pesticides with vegetables and herbs, and (7) not allow use of these pesticides near upstream and public areas. With regard to the types of crops, chlorpyrifos is permitted for use with flowers, field crops, and fruit trees, and paraquat and glyphosate are permitted to be used with rubber, palm, maize, cassava, and fruit trees. Eight Regional Offices of Agricultural Research and Development under Department of Agriculture are in charge of training certified pesticide applicators (8). However, several pesticides other than these three pesticides, are harmful to human health and the environment. Therefore, pesticide applicators should be licensed for all pesticides, not only these three, in order to limit and ensure safe pesticide use.

### 2.6. Promotion of alternative pest management

Alternative pest management should be promoted by the government and relevant organizations in order to reduce pesticide use sustainably. The alternative approaches include Integrated Pest Management (IPM), organic agriculture, Good Agricultural Practices (GAP), agronomic practices, and resistant crops.

IPM is an ecosystem approach to controlling pests by using a combination of techniques such as cultural, mechanical, physical, biological, and chemical control as well as use of resistant crop varieties. The selected pest control approach is the use of methods that minimize risk to human and non-target organisms, while still being environmentally friendly without affecting crop productivity or increasing crop loss (33).

With regard to chemical control, IPM allows pesticide use and combination with other approaches. The criteria for selecting appropriate pesticide use in IPM are as follows: (1) effectiveness against the target organism and low risk of resistance; (2) low acute and chronic toxicity to humans; (3) low toxicity to non-target organisms; (4) fast degradation in the environment; (5) good cost and profit margins for farmers and others (33). The World Health Organization (WHO) and Food and Agriculture Organization of the United Nations (FAO) (34) reported that only 74% of countries worldwide implements the IPM program, 31% implement it throughout and 43% implement partially. In Thailand, the IPM program is implemented in some areas, but is not widespread.

Good agricultural practices (GAP) are a certification system for agriculture in producing safe food for consumers and in accordance with the specified standards. Thai Ministry of Agriculture and Cooperatives established an agricultural standard with Good Agricultural Practices for Food Crops as a voluntary standard in accordance with the Agricultural Standards Act, B.E.2551 (2008) to promote agricultural commodities to meet its standards with regard to quality and safety. There are 8 requirements of GAP for food crops, including (1) water used; (2) planting area; (3) pesticides used; (4) pre-harvest quality management; (5) harvest and post-harvest handlings; (6) holding, moving produce in planting plot and storage; (7) personal hygiene; and (8) record keeping and traceability. The Department of Agriculture is responsible for monitoring and issuing GAP certificates (35).

Organic agriculture is an ecological agricultural system that combines tradition, innovation, and science to sustain the health of the soil, ecosystems, and people. This system employs ecologically based pest control methods, organic fertilizers, and others techniques such as crop rotation and companion planting. Organic agriculture has been established in Thailand since 2009. The Department of Agriculture is responsible for issuing organic vegetable and fruit certificates whereas The Rice Department is responsible for issuing organic rice certificates. The criteria for organic agriculture standards in Thailand are as follows: (1) cultural area located in a suitable environment; (2) no synthetic chemical residues in the agricultural area; (3) no synthetic chemicals in the production process; (4) no chemicals used in seed production; (5) not GMO; (6) no manure used derived from illegal livestock; (7) inputs from the certificate sources; (8) production process does not involve synthetic chemicals; (9) promotion of biodiversity and environmental diversity; (10) officially certified by The Department of Agriculture or Rice Department (36, 37).

The certification system is a valuable system to guarantee safe food for consumers. However, there are several limitations for implementation of this system in Thailand, including a lack of GAP and organic farming knowledge among agricultural officers and farmers, lack of production inputs, a scarcity of markets, complicated and time-consuming monitoring and certification systems, and no database program of certification system for linking the data across all regions (36–38). Although Thai government has attempted to adopt and expand these programs since 1992, there have been no notable success due farmers' ignorance and high costs of pest control (39). Therefore, continual GAP and organic agriculture training for agricultural officers and farmers is important to improve farmers' knowledge and monitor pesticide use implementation process. The government should also support production inputs, technology, and markets as well as develop a monitoring and certification system leading to the long-term sustainability of GAP and organic agriculture (40).

Importantly, there are several labels of certificates attached to the agricultural products in the markets. Organic crops, non-toxic crops, hygienic crops, safe crops, and hydroponic crops are the terms used to describe agricultural products in the markets. In terms of the meaning of each label, organic crops are non-toxic crops in which no chemical pesticides are used in the agricultural process, whereas in hygienic and safe crops pesticides are used in the agricultural process but the pesticides residues are kept to a safe level. The Department of Agriculture certifies hygienic and hydroponic crops, while the Department of Medical Sciences certify safe crops, IFOAM and National Bureau of Agricultural Commodity and Food Standards certify organic crops, and the participatory assurance system certifies non-toxic crops. Unfortunately, fake labels can be seen on agricultural products even if the agricultural products are not certified by any organization and as a result, some consumers are confused about the different types of agricultural products and unsure which labels guarantee safety for them and their family (Table 2) (37). The education of consumers about various agricultural product labels is essential through the media, schools, and communities.

**Table 2:** Classification of agricultural crop labels in Thailand.

**Parameter**	**Organic crops**	**Hygienic crops**	**Safe crops**	**Non-toxic crops**	**Hydroponic crops**	**Chemical crops**
GMO	No	Yes	Yes	Yes	Yes	Yes
Chemical fertilizers used	No	Yes	Yes	No	Yes	Yes
Pesticide used	No	Yes	Yes	No	Yes	Yes
Hormone used	No	Yes	Yes	No	Yes	Yes
Safe for consumers	Yes	Questionable	Questionable	Questionable	Questionable	No
Safe for environment	Yes	No	No	Questionable	No	No
Bio-diversity	Yes	No	No	Questionable	No	No
Certification	Organic agriculture	GAP/none	MRLs for pesticide	PGS/none	GAP/none	None
Organization	-IFOAM -National bureau of agricultural commodity and food standards -PGS	Department of agriculture	Department of medical sciences	PGS/none	Department of agriculture	None

Source: Department of Agricultural Extension, 2016 (37).

MRLs, maximum residue limits; PGS, participatory guarantee system; GAP, Good Agricultural Practices; IFOAM, International Federation of Organic Agriculture Movements; GMO, genetically modified organism.

### 2.7. Promotion of PPE use during pesticide application

In 2015, FAO and WHO announced the International Code of Conduct on Pesticide Management which describes guidelines on the licensing of pesticide applicators. The requirements of PPE during pest control operations are a face shield or full-face respirator; respirators with spare cartridges; long-sleeved coveralls; hats; eye and face protection; chemical-resistant boots; aprons and gloves (41).

Nowadays, the government and associated organizations in Thailand are currently working to educate farmers and farmworkers about the risks associated pesticide use and wearing appropriate PPE during pesticide use. However, it is rather ineffective as expected because of several reasons (11). Previous available studies indicated that Thai farmers and farmworkers used basic PPE during pesticide application, but they never used advanced PPE (specific clothes, waterproof clothes, coveralls, and respirators) during pesticide application. During application, 13.6–97% of pesticide handlers wore a long-sleeved shirt, whereas 56–97% wore long-sleeved trousers, 15.8–97% wore a hat, 43.3–88.4% wore a mask, 8.8–88.3% wore gloves, 7–96% wore boots, and 2.5–87.9% wore goggles (40). The main reason that some pesticide handlers did not wear PPE is hot and tropical climatic weather conditions. Other reasons were a lack of comfort when working in the field, poverty, lack of availability of PPE, and the high cost of PPE (11, 42, 43). They usually wore everyday clothing or whatever clothing was available to protect themselves from pesticide exposure. With regard to respiratory equipment, they usually wore a mask which made of fabric, such as a cotton mask, bandana, or robber mask. These PPE were usually made of woven fabric, therefore were limited in their ability to protect against pesticides effectively. Workers also usually wore a sun hat (44).

A systematic review by Sapbamrer and Thammachai (40) also suggested that the significant determinants associated with PPE use are access to extension services, training programs, information about pesticides, and farm organization. Therefore, agricultural extension services in community are responsible for the education of farmers about pesticide safety practices through a life-long training program. This approach could lead to changes in behavior and raise the awareness of farmers in the long run. The government and associated organizations should implement incentive policies to make PPE more available to farmers, such as lowering PPE prices, expanding the PPE market, promoting domestically manufactured PPE, and lowering PPE taxes.

### 2.8. Strengthening knowledge and raising awareness of pesticide issues

Strengthening knowledge for farmers and agricultural sectors has a major role in changing behavior in reducing pesticide use. Agricultural extensions have a vital role to play in transferring agricultural information and technology to farmers, as well as persuading them to adopt contemporary agricultural techniques. They also serve as a link between agricultural researchers, and farmers (45). In Thailand, The Department of Agricultural Extension has the responsibility and authority for serving and transferring modern agricultural knowledge and technology to farmers across the country. The Department acts as the point of central administration for the making of policy and the dissemination of policy details to locals. The policies of the central administration need to be adopted and implemented by agricultural extensions in provinces, districts, and subdistricts (46). According to a previous report, the existing agricultural extension operation is rather complicated (47). Therefore, government and the central administration should reinforce the function of agricultural extensions by decentralizing to local governments and collaborating more closely with them (48). They also suggested that agricultural extension staff and farmers should be continuously trained to keep up with the changing situation. Previously available studies also suggest that the promotion of health literacy could reduce pesticide use in farmers (49, 50). Therefore, farmers require continual training by agricultural extensions in order to gain new knowledge, access new technology, and understand relevant agricultural issues as well as to raise awareness of pesticide use. Recommended topics for the training course are as follows: (1) effects of pesticides on human health and the environment; (2) costs and expenses associated with pesticide use and medical care in the short and long term; (3) safe use of pesticides for farmers, consumers, and the environment; (4) pesticide application techniques for different types of pest control; (5) alternatives to controlling pests without pesticide use; (6) evaluation and prediction of metrological conditions for crop production; and (7) laws and regulations regarding pesticide use (20, 51).

Strengthening knowledge and raising awareness of pesticide issues in consumers also play an important role in reducing health risks form pesticides. Increased knowledge and awareness with regard to safe food for health and the environment are also essential, as is the education of consumers as to how to choose safe agricultural products from reliable sources and how to read agricultural product labels appropriately (37). Previously available studies in Thailand stated that pesticide residues in vegetable exceed the MRLs. A study in central Thailand found that 42–71% of vegetables from local markets and 35–55% from supermarkets had pesticides exceeding the MRLs (52). A study by Sapbamrer and Hongsibsong (27) also found 13.2% of vegetables from local markets in northern Thailand had pesticides exceeding the MRLs. As a result, information regarding alternatives for cleaning vegetables and fruits in during food preparation, such as the use of sodium chloride, baking soda, and ozone, and other methods is also needed (53, 54). Thai government and relevant organizations have attempted attempting to strengthen knowledge and raise awareness in consumers through a variety of media. However, adoption of participatory programs to change pesticide use behavior among farmers and consumers in the community can raise awareness regarding pesticide toxicity and their risks (55). Therefore, the government, health and environmental sectors, and food industries need to educate consumers regarding the pesticide risks and alternatives to cleaning agricultural products, as well as encourage them to choose and pay for safe food.

## 3. Challenges in implementing government policies

Policy implementation is the process of actually translation a policy into practice in order to address public concern. However, it is frequently seen a gap between policy planning and what actually occurs as a result of a policy (56). There are four key challenges for effective policy implementation in reducing pesticide use and risk in Thailand. First, the current Act does not regulate specific pesticides in agriculture, and illegal use of pesticides has been found. Therefore, the current Act must be updated to reflect current situations (24, 28). Additionally, the government needs to strictly enforce laws, regulations, and legislations. Second, farmers have lack of knowledge and unawareness about pesticide safety practices and alternative pest management. The responsibility of agricultural extensions is to transfer agricultural information and technology to farmers, as well as persuade them to adopt contemporary agricultural techniques (43). However, the existing agricultural extension operation is rather complicated (47). Therefore, the government and the central administration should reinforce the function of agricultural extensions by decentralizing to agricultural extensions and local governments. The government should also clarify the role of agricultural extensions, which are tasked with translating government's policies to farmers, and educating them about pesticide safety practices and alternative pest management. Third, failure in promoting alternative pest management because of lack of production inputs, scarcity of markets, and complicated and time-consuming certification systems (36, 37). Therefore, incentive policies for promoting alternative pest management should be implemented to enhance farmers' compliance. It is important to support funding, production inputs, technology, and markets for farmers who use alternative methods without pesticides. Forth, consumers have lack of knowledge and unaware of safe food for health and the environment. Therefore, the government, health and environmental sectors, and food industries need to educate consumers regarding pesticide risks, and choose and pay for safe food through the media, schools, and communities.

## 4. Conclusion

The government is a key driver to reduce pesticide use and risk. Key challenges for effective policy implementation in reducing pesticide use and risk are as follows: updating specific regulations for pesticides in agriculture; strict enforcement of laws, regulations, and legislations; strengthening of the function and clarifying the role of agricultural extensions, which are tasked with translating government's policies to farmers, and educating them about pesticide safety practices and alternative pest management; implementation of incentive policies for promoting alternative pest management; educating consumers regarding pesticide risks, and choose and pay for safe food. All approaches should be implemented simultaneously and instantly. The government also needs to work together with other sectors, including agricultural sectors, health and environmental sectors, private sectors, as well as food industries. These cooperation results in sustainable problem solving and lower pesticide use and risk in Thailand.

## Ethics statement

This study was exempted by Research Ethics Committee of Faculty of Medicine, Chiang Mai (certificate no. EXEMPTION 9031/2022, Date of approval 2 June 2022).

## Author contributions

Conceptualization, data curation, writing—original draft preparation, supervision, and project administration: RS. Formal analysis and validation: RS, AK, PA, and JP. Investigation, resources, writing—review and editing, and visualization: RS and AK. All authors have read and agreed to the published version of the manuscript.
